# Case-based review of low-field MRI in resource-constrained settings: a clinical perspective from Malawi

**DOI:** 10.1093/bjro/tzaf028

**Published:** 2025-10-14

**Authors:** Karen Chetcuti, Cowles Chilungulo

**Affiliations:** Radiology Division, Department of Paediatrics and Children Health, Kamuzu University of Health Sciences, Blantyre, BT3, Malawi; Blantyre Malaria Project, Kamuzu University of Health Sciences, Blantyre, BT3, Malawi

**Keywords:** low-field MRI, resource constrained settings, low-middle income countries

## Abstract

Low-field MRI (LF-MRI) is in the spotlight as multidisciplinary experts consider it to be one solution to expanding MRI access worldwide. The clinical scenarios and case-mix in which LF-MRI could play an especially important role in the patient diagnostic algorithm are different in High and Low- and Middle-Income Countries (LMIC). The aim of this article is to suggest a robust structure within which to envision clinical use and advancement of LF-MRI technology in LMICs. This article presents three discrete clinical scenarios—a tertiary care facility with an LF-MRI only, a tertiary care facility with an LF-MRI and an HF-MRI and a peripheral healthcare facility with an LF-MRI only—derived from a combination of the authors’ observed practice and hypothetical models in an LMIC and 31 consecutive case reviews within a 32-month timeframe of our experience with the 0.064 T Hyperfine Swoop in Malawi. The authors recognize the important of a holistic approach to the ongoing multifaceted efforts at LMIC-appropriate advancement of LF-MRI technology. This ranges from continued innovation relating to deep learning methods for improved diagnostic accuracy and workflow efficiency, empowerment towards building LF-MRIs in-situ in the LMIC and multidisciplinary capacity building initiatives in LMICs.

## Main

Multidisciplinary discussions amongst global MRI experts have recently taken a new direction, with increasing focus on exploring the role of lower field scanners as a way of addressing the current gross inequity of access to MRI imaging worldwide.

The statistical disparities of MRI unit availability (regardless of their magnetic field strength) globally are huge. In 2025, the International Atomic Energy Agency recorded there being approximately 126 MRI units in low-income countries as compared to 32 814 MRI units in HICs.^1^ (No specific information on the MRI field strength and location of these MRI units (an MRI unit assumed to represent an MRI scanner implemented for clinical use) was found.). The poor representation of MRI in Low- and Middle-Income Countries (LMIC) is likely to be associated with major barriers linked to the implementation of high-field MRI (HF-MRI) units in LMICs including financial challenges; costs at the point of purchase, day-to-day operation and an appropriate and safe environment in which to place an HF-MRI in infrastructurally fragile LMIC radiology ecosystems. Another barrier relates to the paucity of multidisciplinary expertise required to operate an HF-MRI scanner and interpret the acquired scans. LF-MRIs provide an opportunity to address these barriers and therefore to democratize access to MRI as they are portable, more robust, smaller, cheaper and do not require specialist MRI technicians to operate them. From a public health perspective, increasing access to neuroimaging in LMICs; areas with a high burden of HIV, TB and malarial encephalopathies and intracranial non-communicable diseases, is likely to have huge impact on patient morbidity and mortality. The ability of LF-MRI to quantify the burden of intracranial abnormality and the presence or absence of intracranial pathology requiring urgent surgical intervention in each of these diseases is likely to directly impact short and longer-term patient outcomes.

The authors’ experience emerges from The Queen Elizabeth Central Hospital (QECH) in Malawi which is ranked as being a low-income country by the World Bank. The QECH, serves a population of approximately 1 million people.^2^ The QECH was the first sub-Saharan site to acquire and install a Hyperfine Swoop LF-MRI scanner in 2021 with the specifications outlined in Table 1.^3^ While this scanner was originally acquired by one of the research groups for research use, clinicians also piloted its utility as part of routine patient management in a hospital with at the time of installation of Hyperfine Swoop had severe limitations to MRI access. Through our experience in Malawi, we identified at least three clinical scenarios in which LF-MRI scanners are likely to play a critical role in patient management in LMICs.

**Table 1.** Specification of the Hyperfine Swoop (C) LF-MRI Scanner in Malawi.

Feature	Specification
Manufacturer	Hyperfine Inc.
Model	Swoop©
Dimensions: H, W, D (in)	57 × 32 × 57
Field strength (T)	0.064
Supported imaging sequences	Triplanar T1W (standard, gray/white), T2W (standard, fast) and FLAIR, axial DWI with ADC map
Peak power requirement	∼900 w, 15amps, wall outlet
Weight (kg)	∼140
Connectivity	Wi-Fi, ethernet, secure PACS-configuration
Set-up time (minutes)	∼5
Portability	Yes, wheel-based with independent battery

### Scenario 1: tertiary care centre with an LF-MRI and no other cross-sectional brain imaging modalities

Scenario 1 was experienced by the authors in our setting. Over a period of 32-months from March 2022 to October 2024, paediatric hospital clinical teams submitted 43 LF-MRI scan requests. 31 of these patients underwent successful scans. No explanation or written record as to why the other 12 patients were not scanned was found in the paper LF-MRI scan request folder despite efforts at retrospective inquiry by clinical teams. This highlights the common LMIC challenge of incomplete patient records associated with paper trails and no electronical medical record system at our institution during the time of this audit. However, possible reasons for this include patient demise—due to the common scenario of markedly delayed hospital presentation—and patient abscondment which sometimes occurs due to complex background socioeconomic challenges or patients opting for traditional medicine healing pathways. As per an image assessment questionnaire assessing image quality, clinical adequacy and diagnostic confidence, the reporting radiologist rated the scans as good-excellent image quality, having addressed the clinical question posed by the clinicians and having a high diagnostic confidence.

**Figure 1.**
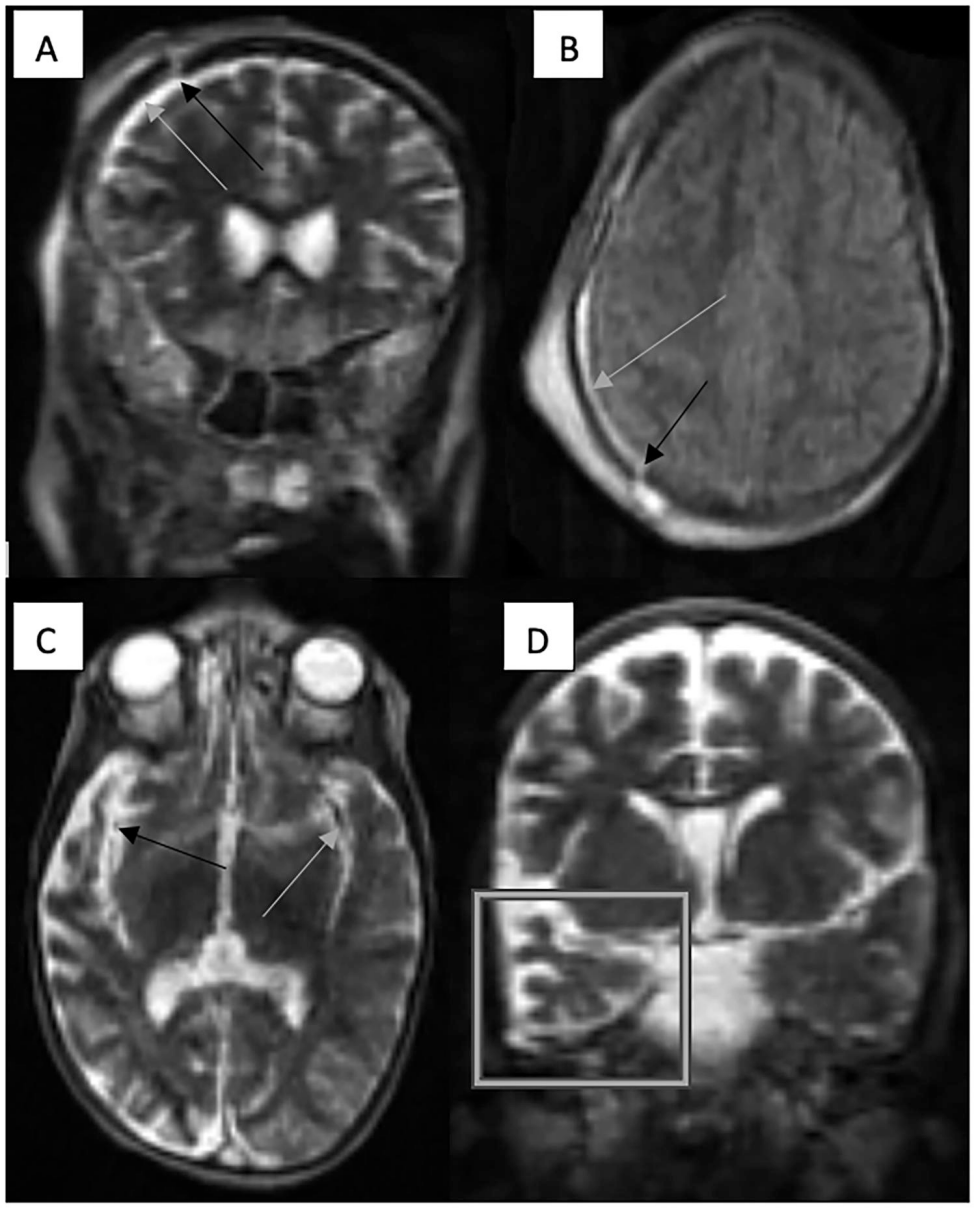
Post traumatic findings in Panels A and B and post ischaemic event sequelae in Panels C and D as imaged on the LF-MRI. Mildly depressed right frontal skull fracture (indicated by the black arrows in Panels A and B) with an overlying scalp oedema/haematoma and underlying widening of the underlying T2W and FLAIR hyperintense subdural fluid space (suspected to be a haematoma) (indicated by the grey arrows in Panels A and B) on the coronal T2W and axial FLAIR sequences. Global atrophy of the grey and white matter in the right temporal lobe (indicated within the grey box in Panel D) on a background of reduced brain volume for the patient’s age with suspected asymmetry with narrowing of the right middle cerebral artery (indicated by the black arrow in Panel C) as compared to its counterpart on the left (indicated by the grey arrow in Panel C) on the axial T2W sequence.

Table 2 outlines the patient demographic, clinical characteristics and the LF-MRI scan requests from the Paediatric Department. Most of the scans were acquired in children aged 1-5 which may relate to the possibility to diagnose intracranial abnormalities in neonates and infants on cranial ultrasound, the wide ability of our clinicians to acquire and interpret cranial ultrasound scans and institutional easy access to portable ultrasound scanners. The majority of scans were of the brain as the Hyperfine Swoop LF-MRI is designed to acquire brain scans. However, scans of the face and neck as regions of interest were also attempted. Several impactful clinical applications of LF-MRIs in LMICs were identified in our experience in this scenario. One example relates to febrile and non-febrile focal neurological presentations; a frequently encountered LF-MRI scan indication in our case set. Multiple clinical differential diagnoses exist for such a clinical presentation and therefore radiology is often critical to the ongoing management of this cohort of patients. The two case examples from Figure 1 which are described below, illustrate how LF MRI has the ability to change patient management in cases in which patients are either not suitable candidates for cranial ultrasound-based diagnoses or unable to gain access to CT or HF MRI as can be extrapolated to the wider LMIC context.

**Table 2.** Request form characteristics and imaging findings of the 31 patients who were scanned on the LF-MRI scanner.

Request form characteristics (*n* = 31)		
**Age**	Neonates (<1 month)	2
	1 - <12 months	3
	1–5 years	12
	6–10 years	4
	>11 years	4
	Not indicated	6
**Sex**	Male	19
**Body part scanned**	Brain	27
	Brain and neck	1
	Face	2
	Neck	1
**Referring departments**	General paediatric	13
	Paediatric intensive care unit	2
	Paediatric neurology	1
	Paediatric neurosurgery	7
	Paediatric nursery	6
	Paediatric oncology	1
	Paediatric surgery	10
	Not indicated	3
**Referring clinical indications**	Complication of hyponatraemia	1
	Intracranial infection	4
	Intracranial tumour	6
	Lymphovascular malformation	4
	Neurodevelopmental regression	2
	Facial tumour	1
	Reduced Glasgow Coma Scale	3
	Seizure ± focal neurology	5
	Suspected clinical syndrome	1
	Suspected ischaemic event	6
	Suspected post-operative brain and neck complication	2
	Suspected post-traumatic intracranial abnormality	2
	Suspected ventricular dilatation/hydrocephalus	6
**LF-MRI findings**	No significant abnormality detected	13
	Cerebral abscess	3
	Lymphovascular malformation: face and neck	3
	Marked cerebral atrophy	1
	Meningitis: bacterial and viral	1
	Non-specific cortical oedema	4
	Facial (orbital) tumour	1
	Porencephalic cyst	1
	Posterior reversible encephalopathy syndrome	1
	Sequelae of stroke; unilateral cerebral hemispheric atrophy and ipsilateral middle cerebral artery narrowing	1
	Subdural haematoma and skull fracture	1
	Tumour: New	1
	Post-operative change	1
	Ventricular dilatation	5

- A 12-year-old male presented with a severe head injury following a fall. He was noted to have a reduced conscious level and was suspected of having a 7th cranial nerve palsy on clinical examination. The paediatric neurosurgical team requested an LF-MRI to characterize the nature and gravity of the suspected intracranial injury. An LF-MRI scan revealed a post-traumatic mildly depressed frontal bone fracture and a shallow right subdural haematoma with no focal parenchymal abnormalities or significant parenchymal oedema. (Figure 1A and B). These LF-MRI findings were critical in directing the patient’s management to a more conservative management as opposed to surgical treatment. This child was treated conservatively and was discharged with no neurological sequelae on clinical review one week later.
- A 4-year-old male presented with a 4-day history of coma and seizures. He had completed medical treatment for suspected cerebral malaria prior to presentation to our hospital. Despite this, he had not recovered from the coma. An EEG on admission was reported as being normal. An LF-MRI scan was requested to identify a structural cause for the patient’s clinical condition. LF-MRI revealed a smaller calibre middle cerebral artery on a background of established and significant ipsilateral temporal lobe atrophy (Figure 1C and D). These findings together with closer clinical history interrogation, were critical in the clinicians’ favouring an acute on chronic condition such as sickle cell disease or an infectious vasculitis rather than acute malarial sequelae in this child. The LF-MRI findings were also impactful by way of exclusion of a space occupying lesion such as a cerebral abscess which would have necessitated urgent surgical management.

It was possible to exquisitely demonstrate millimeter thin intra-ventricular septations identified on intracranial ultrasound and intracranial middle-size vessel calibre discrepancies identified on CT brain angiograms in our small case-set. Further validation studies are required to assess the sensitivity of abnormality detection for LMIC-specific diseases.

### Scenario 2: tertiary care centre with an LF-MRI and a HF-MRI scanner

Increased awareness of the vital role of radiology in the management of patients in LMICs, has resulted in national policy and political agendas in LMICs being more radiologically inclusive. Despite that fact that CT and HF-MRI technology are increasingly found in tertiary care centres in LMICs, there remains a role for LF-MRI scanning technology in these settings for the following reasons:

- The high burden of intracranial pathology and the huge populations served by a single MRI unit (our tertiary centre serves a catchment area of approximately 6 million people), will likely continue to overwhelmingly outnumber the daily scanning capacity of currently available HF-MRI scanners.
- The high infrastructural requirements required by an HF-MRI unit are not easily or reliably available in a LMIC.
- In-country MRI radiographer expertise required to operate an HF-MRI is scarce in a LMIC. Although there may be institutional requirement variations, personnel with no prior MRI technical expertise are able to operate an LF-MRI scanner following a training of short duration (in our case a 1-day training) contributing to sustainability of LF-technology in LMICs.
- Point of care screening and ‘case shifting’ (re-distribution of cases to be scanned on the LF-MRI versus the HF-MRI) with the aim of reducing scan burden on the HF-MRI scanner is another consideration of use for the LF-MRI in the LMIC. Data relating to the clinical demand and expected average daily scans on a HF-MRI scanner in LMICs is limited as HF-MRI are scarce. However, anecdotally, through our experience it is thought that trends in the LMIC would mirror those in the HIC of a high clinical demand further compounded by the high prevalence of intracranial diseases which would benefit from intracranial MRI inclusion in the diagnostic pathway. One clinical example of how the LF-MRI scanner can be used as a screening tool would be the older child with a large or increasing head circumference. The LF-MRI scanner is able to screen the head for ventricular dilatation/hydrocephalus; a common clinical finding in children in the LMIC. The finding of hydrocephalus often necessitates neurosurgical intervention for which post operative serial imaging is required to guide onward neurosurgical patient care. In our experience it is possible to clearly characterize the architecture of complex, septated ventricular dilatation on the LF-MRI. Thus, there emerges an opportunity for LF-MRI as a screening and post operative imaging tool in this patient cohort without the need for the ionizing radiation implicated in CT or the scanning burden and long scanning times associated with HF-MRI imaging.

There is a strong role for portable LF-MRI technology even with the expected increasing presence of HF-MRI technology in LMICs and the known limitations associated with LF-MRIs as discussed in more detail further in the article. Two particular timepoints are thought to be of particular interest for LF-MRI application in LMICs in Scenario 2; screening of patients at their first point of contact with healthcare as well as in their follow-up following the acute clinical presentation. Further implementation research through comparative HF and LF-MRI diagnostic accuracy studies and studies evaluating cost effectiveness of LF-MRI implementation, is required to identify which clinical presentations would benefit the most from use of LF-MRI technology in such a scenario.

### Scenario 3: peripheral healthcare facility with an LF-MRI only

It is highly infrequent to encounter cross-sectional brain imaging tools at peripheral healthcare facilities, which are the often the most geographically accessible entry points to healthcare for LMIC populations. Escalation of patient care through referral pathways to relatively better resourced tertiary care centres is not a decision a clinician working in a LMIC takes lightly, for several reasons. These include the significant distance required to travel on poor and often unsafe road infrastructure and the financial burden on the patient’s family as free hospital to hospital patient transfer is not commonplace in LMICs. In the LMIC, patient transfer decisions are currently largely made on the clinician’s clinical impression with no consistent radiological input available at the point-of-transfer decision. Three examples in which patient outcomes are likely to be improved through the addition of an LF-MRI in diagnostic algorithm of patients being considered for referral from a peripheral healthcare facility to a tertiary care centre in an LMIC are described below.

**Figure 2.**
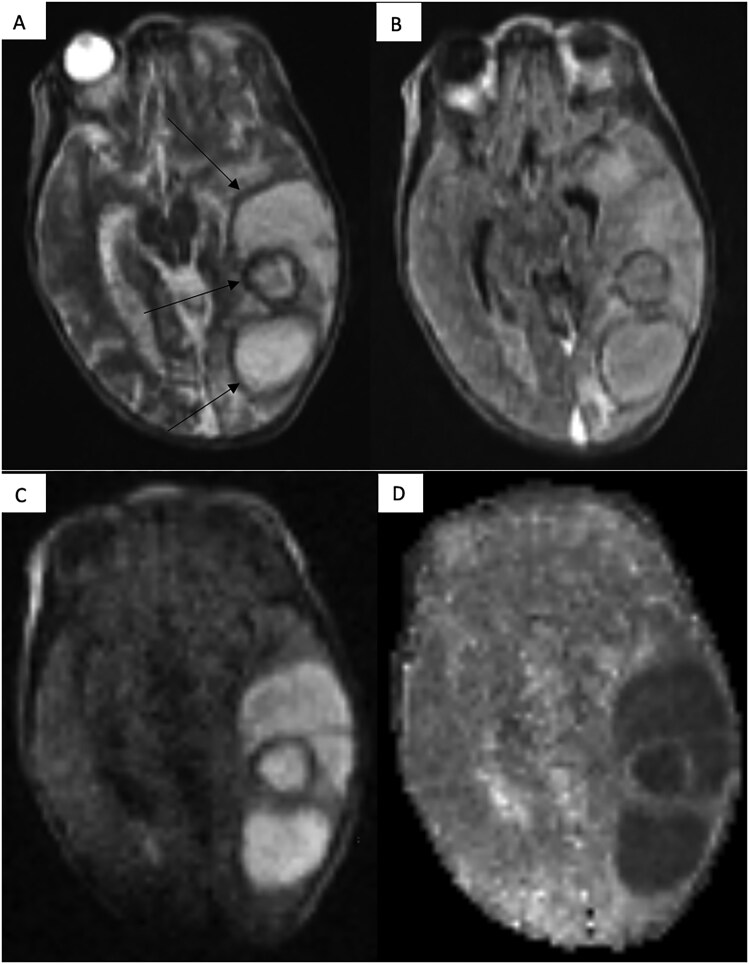
Complex, multicystic left temporal lobe abscess (indicated by arrows) with relatively mild surrounding panrechymal oedema and localised mass effect on the axial T2W sequence in Panel A. Panel B demonstrates the hypointense thick capsule on the axial FLAIR sequence and restricted diffusion within the lesion consistent with pus on the axial DWI 900 sequence and the corresponding ADC map in Panels C and D.

1. The role of LF-MRI in the management of patients presenting to a peripheral healthcare facility with intracranial injuries secondary to a road traffic accident (RTA).
  Clinicians in a peripheral healthcare facility in LMICs most frequently utilise clinical examination findings to determine whether a RTA patient should be referred to a tertiary healthcare facility, based on whether they are likely to have sustained a catastrophic intracranial injury (not amenable to curative surgical intervention) or not. The combination of the varied levels of experience of peripheral healthcare facility clinicians and basing patient transfer decisions on clinical examination findings alone, is challenging. Triaging RTA patients with an LF-MRI at the point of care in the peripheral healthcare facility would significantly empower clinicians.
2. The role of LF-MRI in the management of patients presenting to a peripheral healthcare facility with focal neurological signs and symptoms.
  There is a wide clinical differential diagnosis for patients presenting with focal neurological signs and symptoms. Access to an LF-MRI at the point of care in a peripheral healthcare facility would be crucial in identifying the patient who may benefit from patient transfer to a tertiary healthcare facility as in the case of an intracranial malignancy or infection which would be amenable to urgent treatment in the tertiary healthcare facility (Figure 2). Or, whether the patient is unlikely to benefit from patient transfer to a tertiary healthcare facility in the case of for example an intracranial malignancy that is deemed too advanced, an intracranial infection that can be treated medically at the peripheral centre or in the case of an infarct for which management may be similar in a peripheral or tertiary healthcare facility.
3. The role of LF-MRI in the management of children presenting to a peripheral healthcare facility with a large or increasing head circumference.

LF-MRI at the point of care in a peripheral healthcare facility can confirm/refute an intracranial abnormality and empower the clinician as to the most appropriate onward clinical referral pathway. In the case of the detection of ventriculomegaly, it may be appropriate to enter the patient into a neurosurgical pathway with a view to consideration for a ventricular drain/shunt insertion. Alternatively, in the case of the detection of an intracranial tumour, it would be more appropriate to enter a paediatric oncology pathway. Utilizing LF-MRI as a screening tool is likely to expedite life-saving patient diagnoses and treatment.

The fact that LF-MRIs have significantly lower infrastructural requirements such as the ‘on and off’ electricity requirements that can be powered by solar units, makes their deployment in the peripheral healthcare facility especially attractive and credible. LF-MRI image uploads on secure cloud-based image sharing platforms facilitates referring clinician access to remote radiology expert support.

Utilizing LF-MRI technology as a triaging tool at the point of care at a peripheral healthcare facility is likely to significantly reduce patient morbidity and mortality through timely clinical intervention, improved diagnostic confidence with resultant tertiary care centre patient referrals optimization and shorter in-patient hospital stays with subsequent improved cost-effectiveness.

## Discussion

Further research is needed to address challenges relating to LF-MRI use and implementation which are geographically agnostic as well as challenges which are specific to LF-MRI implementation in the LMIC yet applicable to most of the Scenarios described above. Reduced signal-to-noise ratio (SNR), contrast resolution, sequence acquisition times and motion artefact (the latter two being particularly relevant to the paediatric population) of LF-MRI are location-agnostic.^4^ However, in contrast to work already done in for example Alzheimer’s patients in the higher income setting, there is significant paucity of development and validation research utilizing machine learning methods which target clinical presentations and abnormalities specific to diseases encountered in the LMICs.^5^ Other LMIC-specific LF-MRI implementation challenges relate to the fragile and unstable nature of the LMIC infrastructure inclusive of unstable and unreliable or complete absence of electricity and internet connectivity, particularly in peripheral healthcare settings. The lack of smooth and relative flat flooring with large enough architectural apertures and bedside space significantly challenge the portability feature of current LF-MRI technology in LMICs.^6^ Improved scanner housing and environmental controls are needed. High level of dust pollutant and high temperature conditions often encountered in LMICs are in our experience often postulated to be the cause of equipment breakdown in our setting. The gross shortage of radiologist interpreters is a further significant challenge in LF-MRI implementation in the LMIC as illustrated by the current ratio of radiologists to population being in the order of 1 radiology consultant to a little below 7 000 000 people in Malawi in contrast to approximately 1 radiology consultant to 14 471 people in the United Kingdom.^7^ This can be addressed in two ways. Through further work on in-built automated abnormality detection linked to LMIC-specific point of care questions. Such as work already being done on automated morphometry by Sorby-Adams et al in the niche cohort of patients suspected of having Alzheimer’s disease.^5^ A second way is for LF-MRI manufacturers to allow for robust internet connectivity—to allow for remote radiologist support via tele-radiology—in the design of LF-MRI scanners, especially for those designed for LMIC implementation.^7^ The conceptual proposal of the development of smart triage technology tailored to LF-MRI implementation in the LMIC, in which abbreviated MRI sequence protocols are automatically deployed according to the scan indication, would be particularly impactful in in Scenarios 2 and 3.^8^

LF-MRI offers a significant opportunity to making MRI more accessible globally and could entirely transform patient referral and in-situ acute and elective patient management algorithms in the LMIC. Through its portability, reduced cost, smaller size and its potential to be a powerful triage and screening diagnostic tool as per the authors proposed three-scenario structure and case series illustration from Malawi. Further large-scale validation work in-situ in the LMIC and further research to assess LF-MRI diagnostic utility and cost effectiveness in LMIC-specific diseases is required. Multi-stakeholder collaboration on further optimization of general, geographically-agnostic and LMIC-unique LF-MRI implementation will add to the predicted strong public health role that LF-MRI technology is believed to play in the LMIC.
